# Three discipline collaborative radiation therapy (3DCRT) special debate: The United States should build additional proton therapy facilities

**DOI:** 10.1002/acm2.12537

**Published:** 2019-02-08

**Authors:** Steve Braunstein, Li Wang, Wayne Newhauser, Todd Tenenholz, Yi Rong, Albert van der Kogel, Michael Dominello, Michael C. Joiner, Jay Burmeister

**Affiliations:** ^1^ Department of Radiation Oncology University of California – San Francisco San Francisco CA USA; ^2^ Department of Radiation Oncology University of Texas – MD Anderson Cancer Center Houston TX USA; ^3^ Department of Physics and Astronomy Louisiana State University Baton Rouge LA USA; ^4^ Department of Radiation Oncology West Virginia University Morgantown WV USA; ^5^ Department of Radiation Oncology University of California – Davis Cancer Center Sacramento CA USA; ^6^ Department of Human Oncology University of Wisconsin Madison WI USA; ^7^ Department of Oncology Wayne State University School of Medicine Detroit MI USA; ^8^ Gershenson Radiation Oncology Center Barbara Ann Karmanos Cancer Institute Detroit MI USA

## 1 THREE DISCIPLINE COLLABORATIVE RADIATION THERAPY (3DCRT) DEBATE

Radiation Oncology is a highly multidisciplinary medical specialty, drawing significantly from three scientific disciplines – medicine, physics, and biology. As a result, discussion of controversies or changes in practice within radiation oncology involves input from all three disciplines. For this reason, significant effort has been expended recently to foster collaborative multidisciplinary research in radiation oncology, with substantial demonstrated benefit.^1^, ^2^ In light of these results, we endeavor here to adopt this “team‐science” approach to the traditional debates featured in this journal. This article represents the first in a series of special debates entitled “Three Discipline Collaborative Radiation Therapy (3DCRT)” in which each debate team will include a radiation oncologist, medical physicist, and radiobiologist. We hope that this format will not only be engaging for the readership but will also foster further collaboration in the science and clinical practice of radiation oncology.

## 2 INTRODUCTION

The energy deposition characteristics of protons are substantially different from those of conventional radiotherapy beams of photons or electrons. As a result, the use of proton beams for radiotherapy offers the potential for significant improvements in achievable dose distributions.^3^, ^4^ These differences may result in significant improvements in the efficacy or toxicity profiles of radiotherapy for certain types of cancer. However, such improvements have yet to be demonstrated for many treatment sites. In addition, proton therapy is substantially more expensive than conventional radiotherapy. As such, an important question becomes “How many proton therapy facilities are necessary in the United States?” There are currently 75 operational proton therapy facilities worldwide, with 30 of these in the US alone,^5^ and additional proton therapy facilities are currently both under construction and under consideration. How many such facilities do the science and the economics support?

Arguing for the proposition will be Drs. Steve Braunstein, Li Wang, and Wayne Newhauser. Dr. Braunstein is an academic radiation oncologist at the University of San Francisco – California specializing in the treatment of pediatric and adult primary and metastatic brain and spine tumors. His research focus includes examination of late toxicity in patients undergoing radiotherapy using advanced imaging and clinical analytics toward identification of predictors and mitigation of cognitive impairment and secondary malignancies.

Dr. Wang, is an Assistant Professor at the University of Texas MD Anderson Cancer Center. Her research focuses on radiobiology and radio‐sensitization of tumors of the upper aero‐digestive tract, and assessing the preclinical effects of targeted combination treatments both in vitro and in vivo. Her recent emphasis includes the biological effects of proton vs photon radiotherapy, including relative biological effectiveness, gene expressions, and cell death mechanisms.

Dr. Newhauser, is Professor and Director of the Medical Physics Program at Louisiana State University and Mary Bird Perkins Cancer Center. His research focus is to improve long‐term health outcomes of patients with good prospects for survival of a primary cancer. In particular, his research projects include modeling and measurements of radiation exposures. He researches risk projection, visualization, and optimization, and develops methods and prototype systems to translate these technologies into clinical tools.

Arguing against the proposition will be Drs. Todd Tenenholz, Yi Rong, and Albert van der Kogel. Dr. Tenenholz is currently the Director of Residency Training at the West Virginia University Department of Radiation Oncology. He previously served as the principal pediatric radiation oncologist at Vanderbilt University for 10 yr, and is a member of the Children's Oncology Group.

Dr. Rong earned her PhD in Medical Physics at the University of Wisconsin Madison in 2008. She has been working as a faculty medical physicist for 10 yr and is currently an associate professor in the Radiation Oncology department at University of California – Davis. She has also been serving as a reviewer and Associate Editor for JACMP for more than 8 yr.

Dr. van der Kogel is professor of clinical radiobiology at the University of Wisconsin, Madison. His research has focused on effects of radiation on normal tissues and in particular the spinal cord, as well as on radiation resistance mechanisms related to the tumor microenvironment. He is the recipient of the ESTRO Gold medal and Lifetime Achievement Award, and the ICRU Gray Medal. He is co‐editor of the textbook “Basic Clinical Radiobiology”.

## 3 OPENING STATEMENTS

### 3.A Steve Braunstein, MD, PhD; Li Wang, PhD; Wayne Newhauser, PhD

Photon beam radiotherapy, including x‐rays and *γ*‐rays, is the most widely used type of ionizing radiation in cancer‐directed treatment. Proton beam therapy has emerged over the past several decades as a potentially significantly improved technological advancement for radiotherapy clinical application. Our community of scientists and evidence‐based practitioners in the United States should build additional proton therapy facilities in order to responsibly develop, carefully study, and properly implement this emerging technology such that it may deliver on the promise of improved patient care.

Based upon its advantageous physical features, proton‐based radiotherapy can offer improved dose‐sparing of regional normal tissues while simultaneously allowing for dose‐escalation to the tumor target.^4^ This dosimetric advantage is elegantly achieved by the shape of the Bragg peak; beyond the end of which the dose falls off very quickly.^5^ As a result, the exit dose is but a small fraction of that from photon beam therapies. When this basic physical advantage of finite proton range is utilized with algorithmically optimized treatment planning methods, then delivered with range and fluence modulation, it leads to dosimetrically superior treatment plans, particularly in regions of proximity to uninvolved normal anatomy. Numerous computational studies have predicted lower risks of second cancers and other radiogenic late effects in long‐term survivors who receive proton therapy compared with photon therapy.^6^, ^7^, ^8^, ^9^, ^10^, ^11^ More generally, there is increasing impetus to reduce radiogenic toxicities in normal tissues,^12^ a challenging task common to all types of external beam radiotherapy. Decreased off‐target dose may also engender increased preservation of the immune compartment leading to improved tumor control.^13^

Notably, the technology to deliver proton therapy is significantly distinct from photon‐based delivery and historically has been resource‐intensive, limiting widespread manufacture and deployment of proton delivery facilities. Currently, there are 30 proton centers in operation, 10 centers are under construction or in development, and two centers are expanding in the United States.^3^ Unfortunately, many of these centers are geographically clustered, limiting access to large segments of the population.^14^ Most of the contention regarding further expansion of proton therapy has centered on considerations of absolute cost, cost effectiveness, and related economic considerations. Currently, proton therapy units are the most expensive medical device regulated by the US FDA. Furthermore, the long‐term finances of the US health care system are tenuous and the aging of the population suggest the potential of increased utilization. Thus, cost of care is a very legitimate concern. But to neglect the other costs of cancer in this calculation would be wrong. We have made tremendous advances in cancer treatment outcomes with more than 60% of adults and 80% of children surviving their primary cancer for 5 yr or more (this represents a good surrogate for long‐term disease‐specific survival). Approximately two‐thirds of the cost of cancer to society is attributed to disease‐ and treatment‐related morbidity and mortality. Only about one‐third of the cost goes to direct medical care. Stated in terms of aspirational goals, if we could completely eliminate the morbidity and mortality of cancer, the savings in avoided morbidity and mortality would allow a tripling of the direct medical care expenditures. As decades of medical research progress has shown, an expensive cure is cheaper than an ineffective treatment. With the continued advancement in proton delivery technology, leading to decreased capital and operational costs, the capacity to increase the number of facilities can be realized. Moreover, the greater dissemination of proton facilities enables more investigation leading to refinements in treatment planning, delivery techniques including image‐guidance, and ultimately improved outcomes for select patients. A similar precedent was observed with the widespread deployment and subsequent evolution of intensity‐modulated and volumetric arc based photon therapy, which ultimately emerged as the most advanced iterations of photon technology. The pace of proton therapy development can only be improved with investment in disseminating the technology to more centers.

Based on the above facts, select patients may potentially benefit more from proton vs photon therapy in the respects of normal tissue protection and superior tumor control. Large‐scale collaborative clinical trials and epidemiology studies are needed to determine the role of proton therapy, particularly for children,^15^ and additional treatment capacity is needed to accelerate the accrual of outcome data. The preponderance of available evidence indicates proton therapy is as good as or better than photon therapy for many, but not all, patients. To generate a more complete base of outcomes evidence, more proton therapy centers will be needed to conduct multi‐institutional clinical trials and long‐term epidemiology studies that compare the outcomes of patients who receive proton vs photon treatments. In the end, expanding proton therapy capacity involves risk and uncertainty; there is no guarantee that the centers will cooperate and generate much needed evidence. Conversely, based on more than 6 decades of clinical experience, the desired evidence may never arrive if proton treatment capacity remains at current levels. Therefore, the United States should build additional proton therapy facilities in more states to deliver on the promise of improved patient care.

### 3.B Todd Tenenholz, MD, PhD; Yi Rong, PhD; Albert van der Kogel, PhD

Over the past 12 yr, the United States has seen a dramatic increase in the number of proton therapy centers available to treat patients. In 2006, only four proton centers operated general‐purpose gantries, but since then, an additional 26 facilities have opened, with 10 more centers under construction or in planning. This remarkable growth has occurred in a mixture of settings, with some facilities entirely operated by research‐oriented institutions, while others operate as partnerships with for‐profit entities. At least nine of these centers operate as private‐practice entities with no or minimal ties to an academic research institution. Due to the decentralized nature of the US medical system, all of these centers have developed based on local interest, funding, and philanthropy. With no attempt to build a network of centers that would conduct systematic, nationwide investigation into the potential benefits, possible pitfalls, and knowledge of the infrastructure (both physical and intellectual) needed to utilize this new technology appropriately, much of the data we now have about proton therapy is based on limited, single institution investigations. Few of these studies have direct comparisons between contemporary groups of patients treated with proton vs. photon therapy. This environment has led to a rapid expansion of proton therapy with very limited evaluation of efficacy, and in some cases, with limited evaluation of even the safety of these approaches. Such rapid growth has come at the expense of some “growing pains”.

In 2016, the National Cancer Institute (NCI) convened a national panel to investigate growing concerns about the incidence of brainstem injury in pediatric patients treated with proton therapy.^16^ The growing use of proton therapy as a near mandatory consideration in children with medulloblastoma,^17^ despite the lack of clinical data,^18^ had led to unexpected cases of severe, even fatal brainstem damage. Although first reported in 2014,^19^ the pediatric oncology community had been aware of this problem for several years prior to this initial admission of clinical problems with proton therapy. In comparing the treatment approaches of the three proton centers with the largest pediatric experience, there was a wide range of approaches to treatment planning even among academic institutions, but all of them had calculated effective doses using a fixed relative biological effectiveness (RBE) value of 1.1 for protons. However, the concern that proton RBE for the central nervous system might be higher than 1.1 has recently been confirmed in a comprehensive study in the rat spinal cord.^20^ In this study, the rat cord was irradiated at different positions of the Bragg peak, showing the RBE to increase to 1.2–1.3 at the distal edge. This finding fundamentally disproved the previous assumption of a fixed and uniform RBE, which suggested potential varying degrees of impact on patients that had been or will be planned with a fixed RBE value of 1.1.

As an interim measure, the guidelines of ACNS0831 were modified, essentially allowing dose de‐escalation for patients treated with protons. Subsequent literature 21, 22 has suggested that a more nuanced (and far more complicated) approach to the problem of RBE value in proton therapy planning will be required for clinically accurate modeling of the effect of proton beam therapy on normal tissues.

What lessons can we glean from this experience? The early problems seen in proton treatment of the posterior fossa in children only became widely discussed and acknowledged many years after the first cohort of patients were treated. If these patients had been treated on prospective, dose‐escalation trials, the unanticipated toxicity of the treatment might well have been detected earlier, but would likely have significantly slowed the adoption of proton therapy. By the time these issues came to light, an additional 20 proton facilities had opened, all treating patients with varying techniques. A comprehensive report of the toxicities encountered in this time frame has not been published, and general consensus on the solution to the problem of RBE in proton therapy does not yet exist, much less has been tested widely and made commercially available to private‐practice centers. Is it ethical to continue the expansion of proton therapy when fundamental problems such as the ability to predict toxicity remain unresolved?

From a hospital's sustainability aspect, proton therapy centers are still struggling financially. The Scripps Health Proton Cancer Therapy Center in San Diego opened in 2014, but filed for bankruptcy protection in 2017.^23^ The hope of recouping the initial $220 million investment by treating 2000 patients per year in the San Diego metropolitan area was never achieved. Instead, only about 1400 patients a year have been treated since 2014 according to Scripps.^24^ Most of these patients were treated for prostate cancer. Even such a large volume of relatively simple cases could not keep the center operating on “a break‐even basis”. Based on the 2017 American College of Radiation Oncology Billing and Coding Guide,^25^ 25 fractions of proton radiotherapy can only be billed at the same amount as 44 fractions of intensity modulated photon therapy (IMRT), yet the initial investment for protons is more than ten times higher than photon therapy. This is due to the lack of evidence that would demonstrate to insurers and policy experts that protons have higher effectiveness and better outcome when compared with conventional photon therapy.

From a physics point of view, we are all aware that the proton's famous “Bragg Peak” is a double‐edged sword. It provides sharp dose fall off distal from the Bragg Peak, yet at the same time, it is too sensitive to tumor mobility and patient setup accuracy. Even with stationary tumors and precise patient setup, proton therapy at its early phase (scanning or scattered beam) has not been proven superior to conventional IMRT.^26^ While the more advanced intensity modulated proton therapy technique may be associated with reduced toxicity compared to IMRT,^27^ most reported studies were done in a retrospective fashion, and there is still a dearth of prospective multicenter randomized trials to validate those reported benefits.

One may argue that we need more proton centers to start and participate in those prospective trials. However, there are already two dozen operating proton therapy centers in the United States, while the enrolled patient numbers on the proton arm of numerous prospective trials are still very low. While there may be other issues that are limiting the accrual of patients into prospective trials of proton therapy, the number of proton facilities does not seem to be the problem, and, adding more proton facilities is unlikely to improve accrual.

Overall, we would argue that the unchecked growth in proton treatment facilities is outstripping the radiation oncology community's ability to properly study, analyze, and use this treatment modality. A limited number of proton centers, with a primary mission of research, clinical development, and training would be better able to define the appropriate role and scope for proton therapy. Shortly after the publication of the NCI consensus opinion, the question of expanding proton therapy centers was compared to the development of autonomous vehicles^28^: research and development was not being halted because of “early crashes and technical set‐backs”. Shortly thereafter, such vehicles were placed into “real‐world” use, and then promptly withdrawn after they proved unsafe in this setting. Proton therapy is a powerful tool, but it is clear that we don't fully understand it. It's time to put on the brakes.

## 4 REBUTTAL

### 4.A Steve Braunstein, MD, PhD; Li Wang, PhD; Wayne Newhauser, PhD

We appreciate our colleagues’ thoughtful position against building additional proton radiotherapy facilities. However, while it is true that upfront capital costs of proton facilities are significant in comparison to photon‐based technologies, that cost is decreasing, albeit slowly, with an increasing number of vendors supporting cost‐cutting technological developments in proton therapy.^29^ In addition, though several of the initial proton centers were funded with high profit‐margin expectations, current health care economics have led to adjusted expectations such that future proton facilities are being developed thoughtfully for the current climate, including design of smaller facilities and with community partnerships to ensure sustainability. Importantly, comparisons of true cost‐effectiveness should consider not only the cost of treatment of the primary cancer, but also the actual or estimated costs of late toxicities, which are lower with advanced radiotherapy technologies like proton therapy.^30^, ^31^ Thus, the economic gain of proton therapy will be realized with long‐term follow‐up.

It has taken decades to rigorously study the appropriate parameters for optimum photon‐based radiotherapy delivery, with many clinical, biological, and technical issues still unresolved. The challenges of assessing value with deployment of advanced technologies in radiation oncology, such as protons, are well‐recognized and require a concerted effort of the community to properly study.^32^ Once value is recognized, broader insurance coverage may follow. Such a herculean effort is only afforded by large‐scale cooperative registries and networks of treatment centers of study, demonstrated by the NRG, Alliance, SWOG, and ECOG groups. Large cooperative group studies with focus on proton therapy are emerging but ultimately require more centers to achieve the needed accrual rates. As noted in the opposition statement, the recent effort of an NCI working group addressing the uncertainties in proton therapy RBE and subsequent structured recommendations to minimize radionecrosis risk justify the additional centers to participate in these and other collaborative efforts.

Ultimately, we must acknowledge radiation as an empirical science. After decades of implementation, we are ready to move beyond early phase limited study for proton therapy. No one can deny that the physics and biology of proton therapy can afford more conformal radiotherapy treatment with superior avoidance of normal tissue and thus significantly mitigated toxicity. We are ready to move on to large scale phase III studies, requiring additional proton centers for enrollment to exhaustively examine the parameters for maximum benefit of proton over photon‐based radiotherapy as well as identify new opportunities for improved outcomes. The upfront costs will be readily offset by gains in reduced costs of managing late toxicities. The benefits of proton therapy are established; we do not need to put on the brakes, but rather move forward in a scientific, methodical, and cooperative manner to continue to improve patient outcomes. We owe our patients these efforts and resources.

### 4.B Todd Tenenholz, MD, PhD; Yi Rong, PhD; Albert van der Kogel, PhD

Our colleagues have argued that expanding the number of US proton therapy centers will lead to increased patient access and effectiveness research, and that the direct costs of such expansion are small related to the overall costs of patient care. Unfortunately, while the US has led the world in the adoption of expensive treatment technologies and paradigms, the return on this investment in terms of actual health outcomes has been disappointing relative to other industrialized, English‐speaking countries.^33^ In fact, a great deal of this cost has been transferred to our patients, with over half of US patients diagnosed with a serious medical condition reporting severe financial hardship as a result.^34^ Even if we accept the premise that “an expensive cure is cheaper than an ineffective treatment”, there is little evidence, or even theoretical speculation, that protons will be more effective than photons from a cancer control perspective. While our colleagues may argue that the decrease in late effects promised by protons may justify their cost, the current rapid expansion of proton therapy has not resulted in increased research to establish this argument. As an example, despite enrollment of 437 proton therapy eligible patients in ACNS 0831, only 135 of these patients have been enrolled in the ALET07C1 companion study of neuropsychological outcomes.^35^ This is not a failure of patient access, it is a failure of the treating physicians to prioritize outcomes research.

While there are important arguments to be made regarding the reduction of second primary cancers in the pediatric population, such patients constitute a small minority of patients treated with radiotherapy in the US. While the population of adult 5 yr cancer survivors is growing, these are predominantly patients treated for prostate and breast cancer. The incidence of secondary malignancy in such patients appears to be low in the former,^36^ and has likely been overestimated in the latter.^37^, ^38^ Such arguments hardly justify recent reports of proton therapy for treating small cell lung, pancreatic, and esophageal cancers.^39^, ^40^, ^41^ In fact, the dose limiting toxicities for many adult malignancies relate to tissues in close proximity to the target volume, a situation in which protons may have little dosimetric advantage compared to other treatments, due to high dosimetric sensitivity to internal tumor/organ motion and anatomy change.^42^

In addition to this theoretical advantage of a reduced risk of secondary malignancies, the key argument for the use of protons has been the steep dose fall off beyond the Bragg peak, thus conferring an advantage when treating tumors close to critical normal tissues. As we mentioned in the opening statement, the generally accepted RBE of 1.1 for proton dose delivered in normal tissues has been challenged by the recent rat spinal cord study with a 10% or more increase at the distal edge of the Bragg peak. These uncertainties emphasize the need for (pre)clinical studies of normal tissue tolerance that so far have been lacking. Therefore, the combined impact of dosimetric and radiobiological uncertainties may diminish the claimed benefits of critical organ sparing by proton therapy for various cancer sites.

Our colleagues further offered IMRT and VMAT as an analogy to proton therapy and argued that as more centers adopt this technology, its value will become obvious. However, the well‐established improvement in dosimetric conformity of IMRT over conventional 3D‐CRT planning came at a relatively modest financial cost in the range of 1 million dollars per gantry. VMAT offers a significant, practical advantage of shortened treatment delivery time at even less cost in upgrading the software and hardware. For these reasons, both practitioners and insurers were willing to adopt these evolutions of existing technology based on predicted dosimetric improvements. The dosimetric improvement and potential impact on outcome promised by proton therapy is mostly applicable to a limited population of patients, but the cost of building a proton center is in the range of $100–$200 million dollars.

The current geographic clustering of proton therapy centers is driven by the same market‐driven factors that have led the cost of US healthcare to vastly outpace its improvement in outcomes. The closure of the Indiana University and Scripps proton centers due to financial infeasibility, despite their locations in areas that should have improved accessibility for large populations, highlights the burden that the extreme cost of proton therapy places on the healthcare system and patients. The problem is not access, it is cost. For about $1 million, a cohort of 120 patients eligible for clinical investigation of proton therapy could be given transportation and lodging for the duration of their radiation treatment. This would be far more effective in terms of accomplishing actual clinical research, and orders of magnitude less costly than the construction of a single additional proton facility. Adopting a more “St Jude's” like model for conducting proton therapy research would help to identify the patients who would truly benefit from this new modality, and would likely do so with higher quality data and much lower overall cost than construction of additional proton centers.

## CONFLICT OF INTEREST

No conflicts of interest.
